# Real-time monitoring of *Pseudomonas aeruginosa* biofilm growth dynamics and persister cells’ eradication

**DOI:** 10.1080/22221751.2021.1994355

**Published:** 2021-11-10

**Authors:** Miglė Žiemytė, Miguel Carda-Diéguez, Juan C. Rodríguez-Díaz, Maria P. Ventero, Alex Mira, María D. Ferrer

**Affiliations:** a Genomics & Health Department, FISABIO Foundation, Valencia, Spain; b Servicio de Microbiología, Hospital General Universitario de Alicante, ISABIAL, Alicante, Spain; c CIBER of Epidemiology and Public Health, Madrid, Spain

**Keywords:** *P. aeruginosa*, xCELLigence, biofilms, mannitol, persister cells, antibiotic resistance, ciprofloxacin

## Abstract

Biofilm formation and the appearance of persister cells with low metabolic rates are key factors affecting conventional treatment failure and antibiotic resistance. Using impedance-based measurements, crystal violet staining and traditional culture we have studied the biofilm growth dynamics of 13 *Pseudomonas aeruginosa* strains under the effect of seven conventional antibiotics. Real-time growth quantifications revealed that the exposure of established *P. aeruginosa* biofilms to certain concentrations of ciprofloxacin, ceftazidime and tobramycin induced the emergence of persister cells, that showed different morphology and pigmentation, as well increased antibiotic resistance. Whole-genome sequencing of wildtype and persister cells identified several SNPs, a genomic inversion and a genomic duplication in one of the strains. However, these mutations were not uniquely associated with persisters, suggesting that the persistent phenotype may be related to metabolic and transcriptional changes. Given that mannitol has been proposed to activate bacterial metabolism, the synergistic combination of mannitol and ciprofloxacin was evaluated on clinical 48 h *P. aeruginosa* biofilms. When administered at doses ≥320 mg/L, mannitol was capable of preventing persister cell formation by efficiently activating dormant bacteria and making them susceptible to the antibiotic. These results were confirmed using viable colony counting. As the tested ciprofloxacin-mannitol combination appeared to fully eradicate mature biofilms, we conclude that impedance-based biofilm diagnostics, which permits antibiotic susceptibility testing and the identification of persister cells, is of great potential for the clinical practice and could aid in establishing treatment breakpoints for emerging biofilm-related infections.

## Introduction

Biofilms can be described as bacterial communities immobilized in a self-secreted biopolymeric matrix. This matrix is mainly composed of macromolecules including DNA, proteins and polysaccharides and has a very selective permeability to nutrients and antimicrobial compounds [1,2]. Thus, biofilms have already been described to be up to 1000 times more resistant to antibiotics compared to their planktonic forms and exhibit a large threat to human health [3]. Although bacterial biofilms have been deeply investigated during the past decades, a major limitation is that most existing methods for biofilm research provide information at a single endpoint, therefore missing relevant features about the growth and persistence dynamics [4–6]. In addition, classical biofilm mass quantification usually provides high standard deviations, and can result in lack of reproducibility due to manipulation steps. For this reason, methods for continued monitoring of biofilm growth where the biofilm does not need to be labelled or manipulated have been proposed as a powerful alternative to traditional staining procedures [7,8].

*Pseudomonas aeruginosa* is an opportunistic pathogenic bacterium that causes both acute and chronic infections in severe wounds, urinary tract, or in patients undergoing chemotherapy or any other medical condition related to a weakened immune system like cystic fibrosis [9,10]. The pathogenic success of this bacterium is closely related to quorum sensing systems and the ability to synthesize different metabolites and virulence factors such as pyoverdin, exotoxin A, phospholipase C and elastase [11–13]. Moreover, *P. aeruginosa* infections are extremely difficult to treat, as this bacterium is resistant to many antimicrobial compounds and can easily survive on abiotic and biotic surfaces such as medical equipment even after disinfection [14]. In addition, biofilm formation capacity of *P. aeruginosa* contributes enormously to infection development because biofilms protect this bacterium from immune system attack and conventional antibiotic treatment [15–19].

Another impediment to treat *Pseudomonas*-related biofilm infections is the emergence of persister cells, that are characterized as subpopulations exhibiting a non-dividing state, reduced translation, low proton moving force and decreased ATP levels [20–22]. The appearance of these cells can be triggered by conventional antibiotics or environmental stress, including spatial and nutrient constrains within bacterial biofilms [20,23]. Previous reports have suggested that persister cells exhibit a different phenotype compared to wildtype (wt) strains including smaller colony size or changes in pigmentation [24–26]. Given that persister cells can enter a non-growing state, tolerate high concentrations of bactericidal antibiotic (for example by activating efflux pumps) and regrow once the treatment is ceased, these cells have already been linked to the relapse and recalcitrance of chronic infections [25,27,28]. Although various strategies to combat persister cells such as utilization of DNA crosslinking agents, use of colistin-based antibiotic combinations or addition of glycolysis intermediates have been suggested, there are still many limitations to study and combat the emergence of persister cells within biofilms [29,30].

Thus, the objective of the current work was to study *P. aeruginosa* biofilm formation capacity and growth dynamics, in order to gain insights on persister cell formation and eradication. We tested the biofilm formation capacity of 10 clinical isolates, two reference strains, and the derivative mutant PaO1▵rhl▵las by real-time impedance measurements and conventional biofilm staining methodology. Further, we described the *in vitro* therapeutic efficacy of seven conventional antibiotics on biofilm formation and development as well as the effect of four of them on biofilm eradication once the biofilm was already formed. Using Real-Time impedance monitoring, we were able to identify and study persister subpopulations within mature *P. aeruginosa* biofilms treated with subinhibitory ciprofloxacin concentrations, and to evaluate the effect of mannitol alone and in combination with ciprofloxacin to eradicate the biofilms. Finally, we assessed genetic and genomic differences between dormant *P. aeruginosa* cells treated with ciprofloxacin and controls, in order to identify potential mutations associated with persistence.

## Materials and methods

### Bacterial strains and growth conditions

Clinically relevant *P. aeruginosa* strains were isolated at the Microbiology Department of the Alicante General Hospital (Spain). Model strains ATTC27853 and PaO1, and the derivate strain PaO1▵*rhl*▵*las* (PaO1▵) with a reduced ability to form biofilms, were also used (Table S1). PaO1 was considered as strong, ATCC2753 moderate and PaO1▵ weak biofilm-forming strains, respectively. *P. aeruginosa* strains were cultured on Tryptic Soy Agar (TSA), Lysogeny Broth Agar (LBA) or Brain Heart Infusion (BHI) agar plates at 37°C for 24 h and then inoculated in Tryptic Soy Broth (TSB), Lysogeny Broth (LB) or Brain Heart Infusion (BHI) media at 37°C with vigorous orbital shaking (120 rpm).

### Real-time biofilm growth assay

Real-time biofilm growth monitoring experiments were performed using xCELLigence RTCA SP equipment (Agilent) according to manufacturer’s instructions as previously described [8,31].

To determine the most suitable conditions for *P. aeruginosa* biofilm growth in this system, BHI, LB and TSB media supplemented with or without 0.5% or 1% glucose were tested. Firstly, 100 µl of each medium were used for background measurements. After that, overnight cultures of *P. aeruginosa* were diluted with BHI, LB and TSB growth media supplemented with or without 0.5 or 1% of glucose and 100 µl of these bacterial cell suspensions were added into E-plate wells, reaching a final OD_600_=0.15 and 0.3, respectively. Biofilm growth was then measured every 10 min at 37°C for 72 h.

To evaluate antibiotic efficacy to inhibit biofilm formation, seven antibiotics commonly used in clinical practice were tested: ceftazidime (CAZ, Normon), ciprofloxacin (CIP, Kern Pharma), colistin (CST, Accord), tobramycin (TOB, Brown), imipenem (IPM, Fresenius Kabi), meropenem (MEM, Kern Pharma) and piperacillin-tazobactam (TZP, Accord). Biofilm formation capacity in presence of the antibiotics was monitored for 72 h, in the form of Cell Index (CI) values, which correlate with total biofilm mass [32,33].

For treatment of mature biofilms, antibiotics with highest antimicrobial activity in prevention of biofilm formation (TZP, CAZ, CIP and TOB) were tested. In short, 100 µl of bacterial suspension (OD_600_=0.2625) were used for background measurements. After that, 75 µl of LB were added (reaching the final OD_600_=0.15) and biofilms were grown for 24 h (PaO1) and 48 h (MF120) respectively, depending on the biofilm growth rate of each strain. At this time, corresponding to the highest CIs for both PaO1 and MF120, 25 µl of the corresponding antibiotic were added, reaching final concentrations from 128 to 0.0625 mg/L. Biofilm inhibition/eradication capacity of the antibiotics was then quantified for further 120 h.

### Crystal violet staining

Biofilm formation assay in microtiter plates for crystal violet staining was performed as previously described [5]. Briefly, overnight cultures of *P. aeruginosa* strains were diluted in LB medium to OD_600_=0.1 and 300 µl of bacterial suspensions were transferred into the corresponding wells of 96 well flat-bottom plates 89626 (Ibidi, Germany). Biofilms were grown for 8, 24, 30, 48, 56 and 72 h at 37°C. After that, the culture supernatant was discarded, cells were rinsed using Phosphate Buffer Saline (PBS, pH=7.4) and attached biomass was stained with 0.1% crystal violet (CV) for 20 mins. Subsequently, CV was removed, biofilm-embedded cells were rinsed with PBS in order to remove unattached cells and residual CV and resuspended with 300 µl of 96.5% v/v EtOH. After that, optical density of released CV was measured by means of the absorbance plate reader Infinite M200 (Tecan, Durham NC) at 610 nm.

### Effect of mannitol on planktonic *P. aeruginosa* growth

The overnight cultures of *P. aeruginosa* isolates PaO1 and MF120 were diluted in LB medium to OD_600_=0.1 and 100 µl of these suspensions were added into corresponding wells of 96-well microplates (Thermo Fisher Scientific). After that, 100 µl of mannitol were added into the corresponding wells reaching final concentrations of 3200, 320, 64, 32, 16, 8, 4, 2, 1, 0,5, 0.25, 0.125 and 0.0625 mg/L. Thereafter, microplates were incubated at 37°C with orbital shaking at 120 rpm and the effect of mannitol on planktonic bacterial growth was evaluated for 24 h by the means of an absorbance plate reader Infinite M200 (Tecan, Durham NC).

### MIC determination

The minimum inhibitory concentrations (MICs) of CIP, CAZ, COL, TOB, IMI, MEM and TZP were assessed using the CLSI broth microdilution method as previously described [34]. UMIC microdilution test was used for CST (Biocentric, Bucker) and E-tests (Biomérieux) were used for the rest of antibiotics. MBICs (minimum biofilm inhibitory concentrations) were calculated for PaO1, and for the clinical isolates with the strongest biofilm formation capacity (i.e. MF120 and MF124), considering the time points where antibiotic-free cell control reached the highest CIs (24 h for PaO1, 48 h for MF120 and 72 h for MF124, respectively), following Ferrer et al. [8].

### Identification of persisters and regrowth assay

To confirm the presence of bacterial persistent cells and not CIP resistant mutants, *P. aeruginosa* MF120 biofilms were cultivated in the xCELLigence system for 48 h as described above. After that, CIP was added, reaching a final concentration of 0.25 mg/L. At 140 h of biofilm growth, the experiment was stopped, and the biofilms exposed to CIP were plated on LBA plates containing 0.25 mg/L of CIP, in order to maintain the persister phenotype, while MF120 control cells were plated on LBA plates without additional antibiotics.

Besides the changes in growth, the ability of white persister cells (MF120 treated with CIP) to revert to greenish wt phenotype was investigated by two independent observers. Five persister colonies and five controls without antibiotic were selected and resuspended in 200 µL of LB media containing mannitol (3200 mg/L) and LB alone and grown at 37°C for 120h to observe the switch from persister population into actively growing cells.

### Effect of mannitol alone and in combination with ciprofloxacin on *P. aeruginosa* persisters

To describe the effect of mannitol on *P. aeruginosa* biofilm formation, mannitol was serially diluted in LB medium reaching final concentrations of 3200–0.0625 mg/L. One hundred microliters of each dilution in triplicate were used for background measurements. After that, 100 µl of PaO1 and MF120 bacterial suspensions were added into the corresponding E-plate wells reaching final bacterial cell optical density of OD_600 _= 0.15 and continuous biofilm growth was observed for 72h in xCELLigence equipment.

The ability of mannitol to potentiate the effect of CIP and/or prevent persister bacteria formation within *P. aeruginosa* MF120 biofilms was then investigated using mannitol concentrations of 3200, 320, 64, 32 and 16 mg/L. Firstly, mannitol was added together with bacterial inoculum (OD_600_=0.15). After 48 h of biofilm growth, 25 µl of CIP were added (final concentration: 0.25 mg/L), and biofilm growth was observed for additional 140 h. Similar experiments were performed adding 25 µl of mixed suspensions of mannitol and CIP on mature MF120 biofilms at 48 h. Biofilm growth after addition of both compounds was quantified for 140 h.

### Colony forming unit (CFU) counting

To assess the number of viable cells after biofilm treatment with mannitol, CIP or their combination, *P. aeruginosa* biofilms were collected at 140 h of growth in the RTCA system and sonicated for 5 min in order to eliminate bacterial aggregates and disrupt extracellular biofilm matrix. After sonification, serial dilutions were prepared, plated in triplicates on LB plates and incubated at 37°C overnight. CFUs then were counted, averaged, and expressed as log10 CFUs. Each experiment included three technical replicates and was repeated three times (biological replicates). Statistical significance was assessed using Student’s *t*-test, where *p-value* 0.05 was considered as significant.

### Whole genome-sequencing and bioinformatic analysis

DNA from individual colonies grown in the presence of CIP (0.25 mg/L), mannitol (3200 mg/L) or their combination (exhibiting non-identical phenotypes) was extracted using MagNA Pure LC DNA Isolation Kit III for Bacteria and Fungi (Roche Diagnostics) following manufacturer’s instructions. For each colony type, two colonies from the same agar plate were selected as replicates. After that, Illumina libraries were constructed at the Sequencing Platform in FISABIO (Valencia, Spain) and the genomes were sequenced with NextSeq technology (Illumina) using the 500/550 High Output 75 cycles Kit (single-ends 75 bp reads).

Resultant sequenced reads (75 bp long) were trimmed for low quality reads (<20) and length (<50) using prinseq [35]. SPAdes [36] was used for genome assembly and ORFs were detected using prodigal [37] and annotated using hmmsearch [38]. For genomic mutations, the software flex2 (https://github.com/asierzaragoza/flex2) was used to visually detect genomic insertions, duplications or inversions. Single-nucleotide polymorphisms (SNPs) were detected using the software snippy and snippy-core through the Galaxy server (https://github.com/tseemann/snippy). The assembled contigs and the original reads have been deposited in SRA database under the accession number PRJNA753320.

### Statistical analysis

Biofilm inhibition/induction after treatment with different antibiotics were considered to be significantly different from controls using linear models from the lm library in the R Statistical Package version 1.0.1.7 with *p*-value <0.05 (https://cran.r-project.org/web/packages/glmulti) (accessed in January, 2021). Student’s *t*-tests were performed to reveal statistical differences between treatments after CFUs counts. *p-values* < 0.05 were considered significant.

## Results

### Influence of culture conditions on *P. aeruginosa* biofilms

Biofilm assembly can be influenced by many different factors including culturing conditions and initial inoculum size [8,31]. We investigated the effect of initial optical density (OD) and culture growth medium on bacteria attachment and biofilm formation on E-plate surfaces. Figure S2 shows biofilm formation dynamics of model strain PaO1 and clinical isolates MF117, MF118 and MF124 when grown in TSB media using initial ODs_600_ of 0.15 and 0.3. Results showed only slight differences in total biofilm mass (CIs) for all the tested strains. In addition, we tested how biofilm growth dynamics might be influenced by several growth media: LB, TSB and BHI. Since CIs reached with BHI media with or without additional glucose were very low (between 0.01 and 0.1) for most tested strains compared to other culture media, this growth medium was considered as not suitable (data not shown). On the contrary, both TSB and LB media permitted *P. aeruginosa* to form robust biofilms overtime, reaching high biofilm mass (CI values up to 10 times higher when compared to those observed using BHI medium) (Figure S1). The use of TSB medium (with and without additional sugars) resulted in biofilm growth delay for all tested strains, except clinical isolate MF123 (Figure S1ab). For example, strain PaO1 reached the maximum CI in LB medium at 24 h, while similar CI was observed only at 55 h in TSB-0.5%-glucose. A similar trend in biofilm growth delay was observed when LB medium was supplemented with 0.5% of glucose (Figure S1d). Given its highest biofilm formation capacity, LB without additional glucose was chosen for *P. aeruginosa* biofilm growth in the xCELLigence system.

### Comparison of impedance-based measures to CV staining

**Figure 1.**
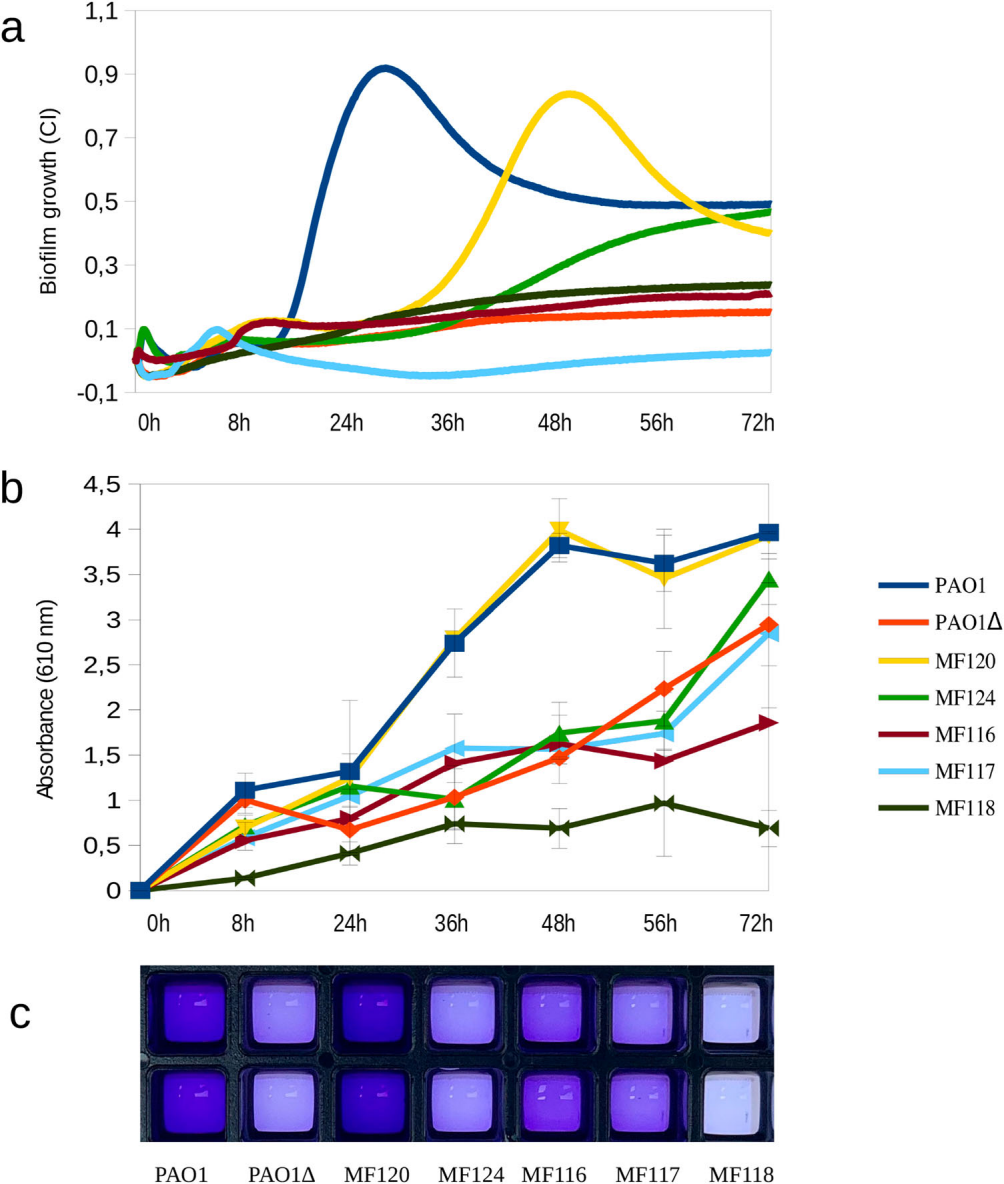
Biofilm formation capacity of seven *P. aeruginosa* strains as quantified by impedance-based measurements in xCELLigence (a) and by Crystal Violet (CV) staining (b, c). Panel **b** represents the absorbance of released CV at 0, 8, 24, 36, 56 and 72 h, where higher absorbance indicates larger biofilm growth. Panel **c** depicts biofilm formation in ibiTreat 96-well plates at 36 h, where more intense colour reflects larger biofilm mass. Bacterial strains used in the experiment are indicated in the legend and include a mutant of PAO1 strain with impaired biofilm formation. Data are the means of three biological replicates.

Culturing plates materials have an impact on the adhesion capacity of *P. aeruginosa* and estimates of biofilm mass are also influenced by the quantification method used [39]. Figure 1(a) represents biofilm growth dynamics of different *P. aeruginosa* strains when grown in 96-well E-plates, as measured by impedance during 72 h; panel b corresponds to the quantification of released crystal violet measured as OD_610_ at 8, 24, 36, 48, 56 and 72 h, while panel c depicts *P. aeruginosa* biofilm biomass staining with crystal violet at 36 h of growth for each tested strain. The results indicate that most tested strains had a similar biofilm formation and growth dynamics in both xCELLigence and Ibidi plates, suggesting that these methodologies are comparable. For example, strains PaO1, MF120 and MF124 were the strongest biofilm formers in both settings. However, as it can be seen in panel b, some of the isolates were able to adhere and form biofilms faster or stronger in Ibidi plates.

### Effect of conventional antibiotics on biofilm formation

For further analysis, we chose *P. aeruginosa* strains that exhibited the highest biofilm-forming capacity as measured by impedance (PaO1, MF120 and MF124) and evaluated the effect in real time of seven conventional antibiotics commonly used in clinical practice (ciprofloxacin (CIP), tobramycin (TOB), ceftazidime (CAZ), colistin (CST), piperacillin-tazobactam (TZP), imipenem (IPM) and meropenem (MEM)).

**Figure 2.**
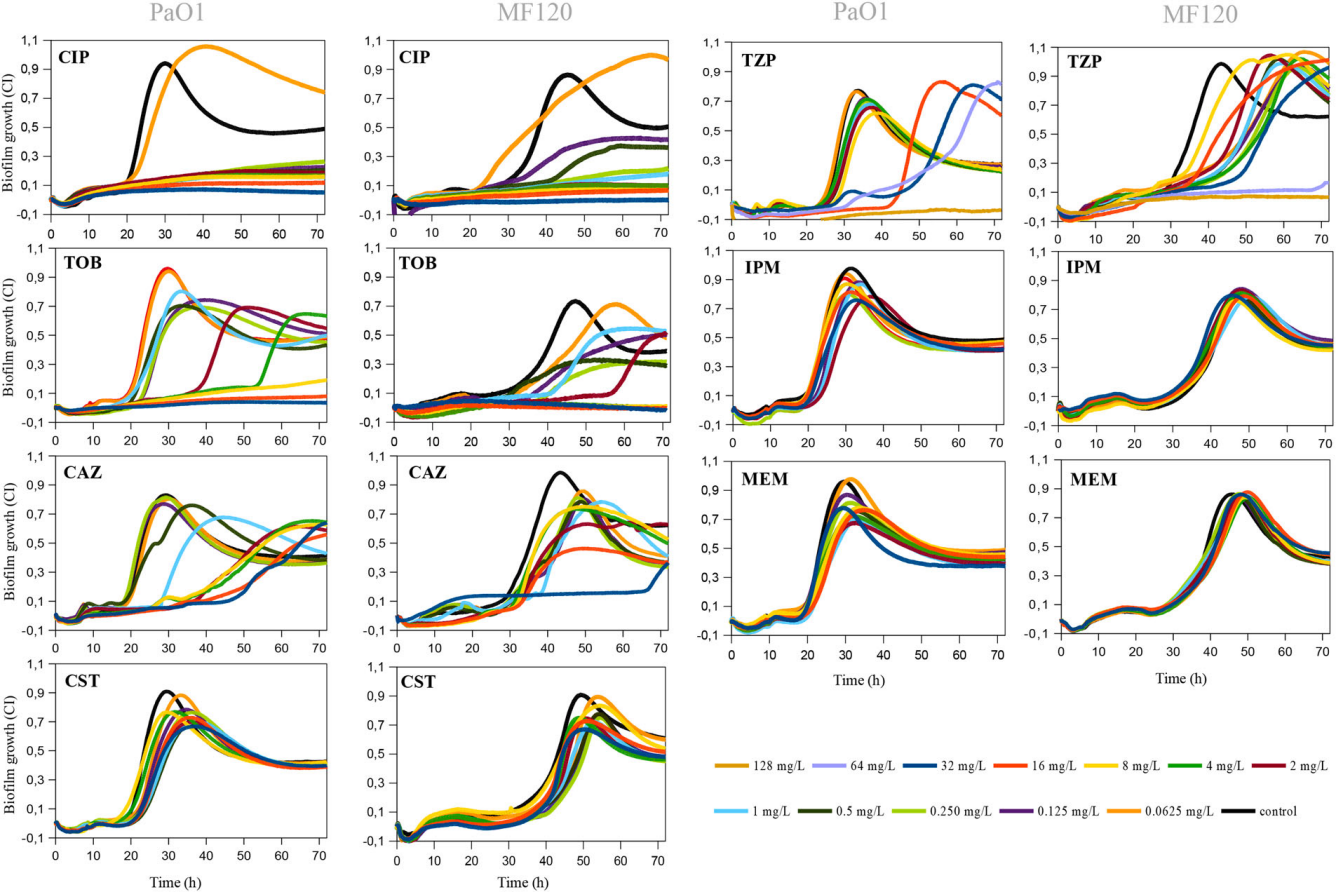
Effect of ciprofloxacin (CIP), tobramycin (TOB), ceftazidime (CAZ), colistin (CST), piperacillin-tazobactam (TZP), imipenem (IPM) and meropenem (MEM) on *P. aeruginosa* PaO1wt and MF120 biofilm formation. Graphs show estimates of total biofilm mass as measured at 37°C using impedance-based measurements. Black lines indicate untreated controls. Each line represents the mean of three biological replicates. All antibiotics were added at the beginning of the experiment together with bacterial inoculum from 0.625 to 32 mg/L for all antibiotics except TPZ (0.625 to 128 mg/L). SDs are not shown for clarity.

Figure 2 summarizes the effectiveness of antibiotics on biofilm formation in PaO1 and MF120 strains, while Figure S3 depicts the effect of these antibiotics on MF124. CIP showed a high efficiency and suppressed biofilm formation of all tested strains in a dose-dependent manner. However, the exposure of PaO1 and MF120 biofilms to 0.0625 mg/L of this antibiotic resulted in biofilm growth induction and changes in biofilm growth dynamics (Figure 2). TOB and TZP also inhibited biofilm formation when added at high concentrations. In addition, lower concentrations were not able to inhibit biofilm formation for any of the tested strains, although most of them resulted in biofilm growth delay or decreased biomass. Although none of the tested concentrations of CAZ could fully inhibit biofilm formation in any of the tested strains, the inhibitory capacity of this antibiotic was concentration-dependent, and most concentrations of CAZ resulted in biofilm growth delay. Neither CST nor IPM or MEM could completely inhibit or delay biofilm growth in PaO1 and MF120. In contrast, the exposure of MF124 biofilms to 32 mg/L of CST resulted in complete biofilm growth inhibition, while lower concentrations of CST showed a dose-dependent effect (Figure S3). These results indicate that each biofilm-forming strain should be analyzed individually, taking into account more than one end-point in order to assure the best clinical outcome.

Finally, Table 1 shows antibiotic susceptibilities of planktonic broth cultures (MIC) compared to the same isolates when they were grown in biofilms (MBIC). MBICs were up to 32 times higher compared to traditional MICs obtained by E-test and microdilution (Table 1 and Table S2).

**Table 1.** Minimum inhibitory concentration (MIC) vs minimum biofilm inhibitory concentration (MBIC) values for ciprofloxacin (CIP), tobramycin (TOB), ceftazidime (CAZ), colistin (CST), piperacillin-tazobactam (TZP), imipenem (IPM), and meropenem (MEM) in different *P. aeruginosa* strains.

Minimum Inhibitory Concentration/Minimum Biofilm Inhibitory Concentration							
Strain	CIP	TOB	CAZ	CST	TZP	IPM	MEM
PAO1	0.094**S**/0.125	1.5**S**/2	1**S**/2	1**S**/>32	3**S**/16	1.5**S**/>32	0.38**S**/>32
MF120	0.125**S**/0.250	<=2**S**/4	2**S**/16	1**S**/>32	<=8**S**/32	<=1**S**/>32	<=1**S**/>32
MF124	0.19**S** /2	1**S**/8	2**S**/>32	1**S**/32	8**S**/32	**2S**/>32	0.75**S**/>32

Notes: MICs were measured by standard protocols (UMIC microdilution test for colistin and E-tests for rest of antibiotics) and expressed as (mg/L), while MBIC were calculated according to impedance-based measurements in xCELLigence system. S and R in the table indicate if a strain is considered susceptible or resistant according to EUCAST guidelines [34]. MBICs were assessed by the evaluation of normalized biofilm growth curves using impedance measures (Cell Index measurements) at 24 h for PaO1, 48 h for MF120 and 72 h for MF124, respectively, following Ferrer et al. [8].

### Capability of antibiotics to eradicate pre-formed biofilms

**Figure 3.**
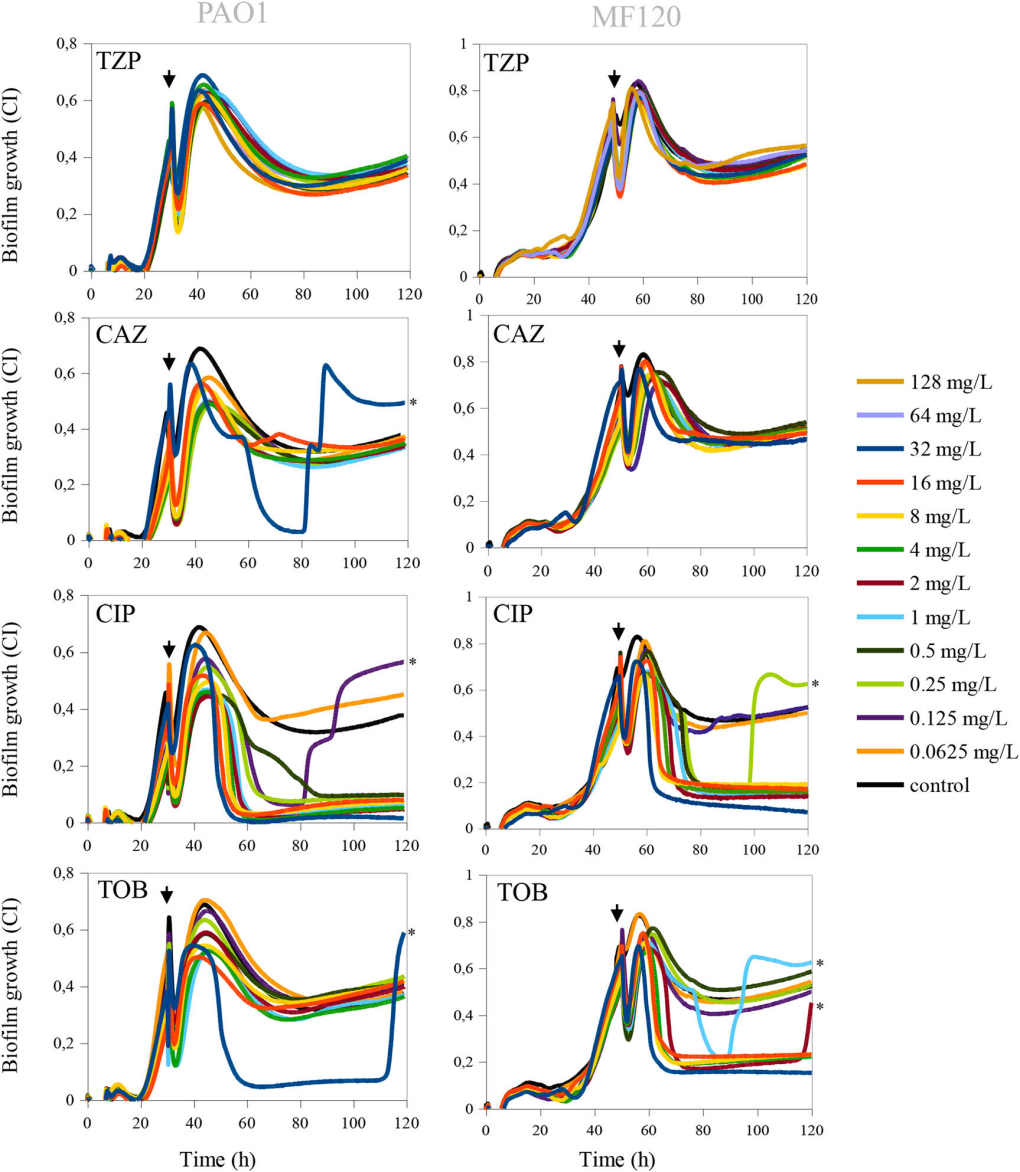
Effect of piperacillin-tazobactam (TPZ), ceftazidime (CAZ), ciprofloxacin (CIP) and tobramycin (TOB) on mature biofilms of PaO1 (24 h after inoculation) and MF120 (48 h) strains. After the addition of the antibiotics, biofilm growth was observed for additional 100 h. Black lines represent antibiotic-free controls while black arrows indicate the time when antibiotics were added. Each line represents the mean of three replicates. The emergence of dormant, persister cells, which appeared after the biofilm eradication using different antibiotics, is marked by black asterisks.

Pre-formed biofilms are notoriously difficult to eradicate, as antibiotics must cross the biofilm matrix to reach biofilm-embedded bacteria [40]. Thus, to obtain further insights on biofilm eradication dynamics, we chose the antibiotics that showed the highest efficacy when added at the beginning of biofilm growth (TZP, CAZ, CIP, TOB) and tested their ability to eradicate *P. aeruginosa* PAO1 and MF120 biofilms at 24 and 48 h, respectively. Impedance-based measurements showed that the tested concentrations of TZP (128–0.0625 mg/L) could not disrupt biofilms of the examined strains (Figure 3). CAZ, which has a similar mechanism of action to TZP and inhibits bacterial cell wall synthesis, had no biofilm inhibitory effect on MF120 biofilms, but was able to disrupt PAO1 biofilms when added at the highest dose tested (32 mg/L). However, the increase in CI at 90 h suggests that this antibiotic is not able to kill all biofilm-embedded bacteria, which could consequently result in relapsed biofilm infection (Figure 3). On the other hand, when mature *P. aeruginosa* biofilms were exposed to CIP, high concentrations of this antibiotic suppressed new biofilm accumulation and completely eradicated the pre-formed biofilm in both PaO1 and MF120 strains. On the contrary, low concentrations of this antibiotic (0.0625 mg/L in PaO1 and 0.125–0.0625 mg/L in MF120) were ineffective. In addition, we observed that certain concentrations of CIP (0.125 mg/L in PaO1 and 0.25 mg/L in MF120), although initially seemed effective, resulted in a sudden increase in CIs at approximately 80 and 100 h, respectively. This second peak of growth observed after treatment with these CIP concentrations suggested the emergence of a dormant cell fraction within PaO1 and MF120 biofilms (Figure 3), which was later investigated in more detail.

Comparably to CIP, TOB completely eradicated mature MF120 biofilm when added at relatively high concentrations (32–4 mg/L), suggesting that TOB can penetrate through the exopolysaccharide matrix and efficiently kill biofilm-embedded bacterial cells. On the other hand, TOB was not efficient on mature PaO1 biofilm, given that none of the tested concentrations could disrupt the biofilm, indicating a strain-dependent effect. In addition, similarly to CIP, TOB at 1 and 2 mg/L resulted in the rise of a second biofilm peak at 90 and 120 h, respectively, suggesting that both antibiotics can only kill a part of bacterial cells embedded in the biofilms and induce the emergence of dormant cells within *P. aeruginosa* biofilms.

### Mannitol effect on planktonic and biofilm growth

**Figure 4.**
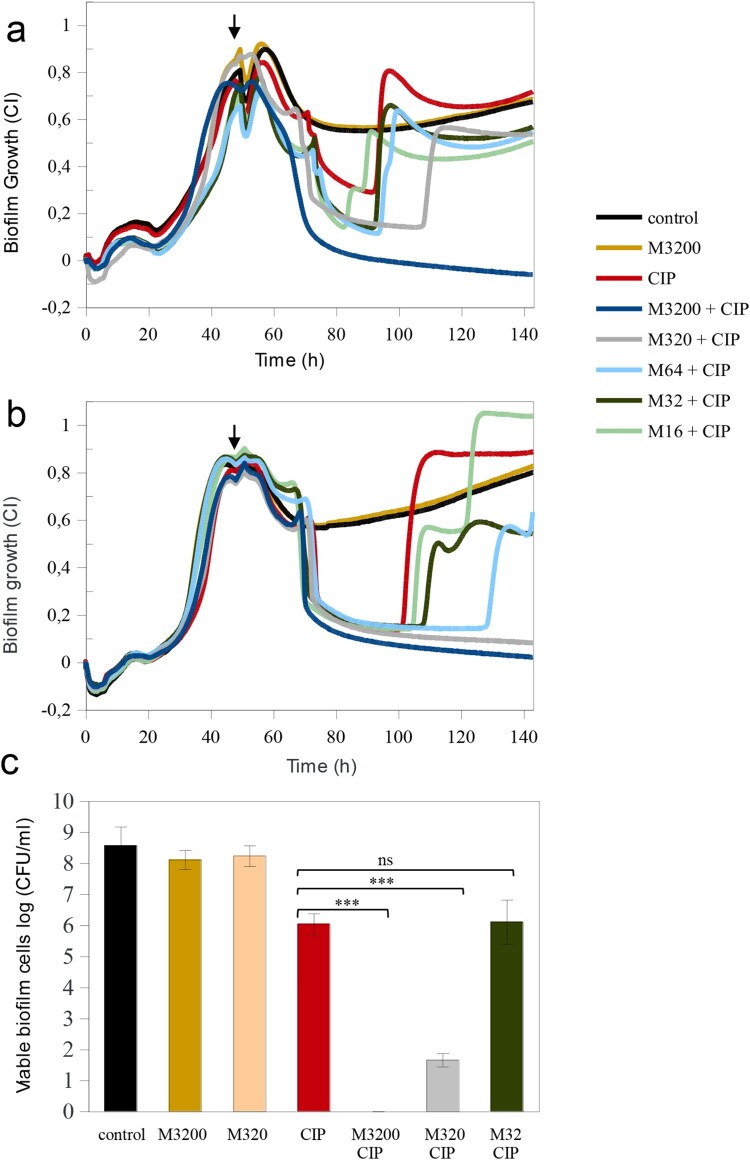
Mannitol effect alone and in combination with ciprofloxacin in the eradication of persistent *P. aeruginosa* MF120 cells. (a) Biofilm growth when mannitol was added at the beginning of the experiment, and ciprofloxacin added at 48 h (vertical arrow). Panel (b) corresponds to a second experiment where both mannitol and CIP were added together at 48 h (vertical arrow), reaching final concentrations of 3200–16 mg/L for mannitol and 0.25 mg/L for CIP. Panel **(c)** represents bacterial cell viability after treatment with different combinations of mannitol and CIP. Data show the average of log CFUs counts from three biological replicates. Statistical significance was assessed by *t*-test at 140 h; asterisks indicate *p* ≤ 0.001, ns–not significant. M–mannitol; CIP–ciprofloxacin (0.25 mg/L).

A range of concentrations of mannitol were tested to characterize its effect on both planktonic and adherent *P. aeruginosa* growth. Absorbance measurements showed that the tested concentrations did not affect PAO1 or MF120 planktonic growth (data not shown), indicating that this compound does not have antimicrobial activity. Given that many compounds without antimicrobial effects have been linked to biofilm disassembly [41–43] we have also tested if mannitol had an effect on biofilm disaggregation. Impedance measurements showed that the tested concentrations did not affect biofilm mass compared to the untreated control (Figure 4(a)). This suggests that mannitol alone is not a biofilm dispersal agent. Thus, considering that mannitol has been shown to be involved in the switching of persister and tolerant phenotypes to metabolically active cells [44], we further evaluated how the combination of mannitol and CIP could affect biofilm formation and maturation of pre-formed MF120 biofilms in real-time.

### Mannitol enhances the effect of ciprofloxacin

The addition of mannitol alone at the beginning of biofilm growth (3200 mg/L) did not result in any changes in total biofilm mass compared to the untreated control (Figure 4(a)). However, when mannitol pre-treatment (3200 mg/L) was combined with a CIP concentration that favoured the appearance of dormant cells (0.250 mg/L) added on a 48 h biofilm, the biofilm was completely eradicated. Given that lower mannitol concentrations were not efficient and resulted in the appearance of a second biofilm peak, we conclude that mannitol may be metabolized overtime. For this reason, we have tested whether mannitol could potentate CIP effect more efficiently when added together with the antibiotic at 48 h. Figure 4(b) shows that the addition of 3200 or 320 mg/L of mannitol, in combination with CIP at 48 h resulted in a complete disruption of mature MF120 biofilms. However, the combination of CIP with lower mannitol concentrations was not sufficient to revert all persister cells into actively growing bacteria. Nevertheless, a concentration-dependent effect was observed, with a delay in the second growth peak at mannitol concentrations of 16, 32 and 64 mg/L. These results were confirmed by colony counts (Figure 4(c)), where CIP alone reduced cell viability 2.5 orders of magnitude, whereas 3200 mg/L of mannitol combined with CIP completely killed all bacteria embedded in the biofilm matrix while 320 mg/L resulted in almost seven orders of magnitude reduction in viable cell number (*p-value*<0.0001).

### Identification of persister subpopulations

**Figure 5.**
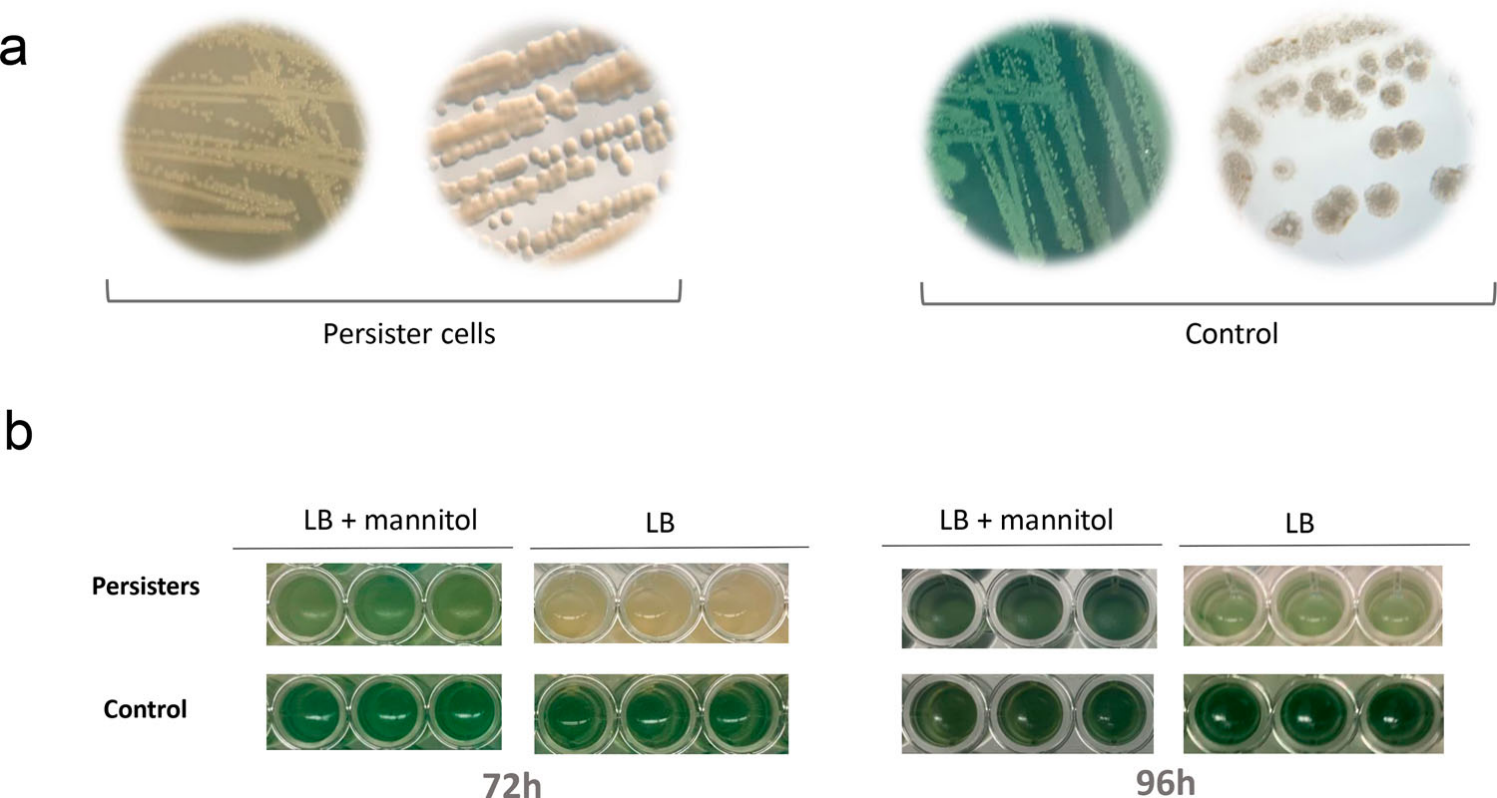
Persister regrowth assay. (a) Phenotypical differences between persister cells (treated with ciprofloxacin [CIP] 0.25 mg/L) and control cells. Clear differences in pigment colouration are observed. In order to maintain persisters phenotype, MF120 cells treated with CIP were subcultured in LBA plates supplemented with CIP, while controls were continuously cultured in LBA plates without antibiotics. (b) Persister regrowth and reversion into actively growing cells in fresh LB media supplemented with mannitol (3200 mg/L) or LB alone at 72 and 96 h, respectively. Three biological replicates and negative controls for each condition were included in the experiment. Pigment reversion phenotypes are observed with time in persister cells, and the reversion is accelerated in the presence of mannitol. The genomes of control and persister cells were fully sequenced and the observed mutations are described in Table 2.

Bacterial cells grown with CIP after the second CI peak were cultured on LBA plates with CIP in order to maintain the persister phenotype. Persister cells had a different colony morphology, were less mucoid and were not able to produce pyocyanin (white colonies). In addition, they were opaquer, compared to the greenish untreated control colonies plated on LBA plates without additional antibiotics (Figure 5(a)).

It is known that persisters revert to a normal wt phenotype once antibiotic presence is ceased [45]. Thus, we further determined the reversibility of this phenotype after CIP removal. Persisters and untreated controls were reinoculated into fresh growth media supplemented with mannitol (3200 mg/L) and without mannitol (LB alone) and observed bacterial growth and colour changes. After 72 h of growth, persister cells had a phenotype similar to that of untreated controls in media which contained mannitol but not in LB alone, concluding that persisters were reverted into actively growing cells. In addition, persister cells that were inoculated in LB media without mannitol restored pyocyanin production at 96 h (Figure 5(b)) concluding that mannitol accelerated the reversal of persisters to the wt phenotype. We have also assessed MICs for persister cells subcultured in LB agar plates with CIP and found that the resistance to this antibiotic was increased at least 10 times. Persister cells had also increased resistance to IMI and MEM (Table S3). Finally, we reinoculated persisters to fresh LB media, LB media supplemented with mannitol and LB media supplemented with CIP. MICs performed at 96 h of inoculation showed that persister cells grown in the presence of mannitol became more susceptible to most of the tested antibiotics, while in LB alone they showed increased resistance to CIP and IMI. This indicates that, in addition to phenotypic changes (Figure 5), persisters grown in the absence of antibiotic also revert their resistance profile. On the contrary, persisters reinoculated in LB media supplemented with CIP maintained the white phenotype and showed increased resistance to all the tested antibiotics (Table S3).

### Genetic changes associated with the persister phenotype

We extracted DNA from colonies grown in the presence of CIP (0.25 mg/L), mannitol (3200 mg/L) or their combination that represented non-identical phenotypes and sequenced their genomes in order to test if phenotypic differences were caused by genetic mutations (SNPs, insertions, deletions or inversions). For each colony type, we selected two colonies from the same agar plate as replicates. In particular, the colonies had the following phenotypes: CIP were small and white (isolates 7, 8) and (isolates 9, 10) big white; mannitol (isolates 13, 14) were green, and their combination (isolates 11, 12) were small white. In addition, these phenotypes were compared to green (isolates 3,4) and white (5, 6) colonies of untreated controls and to the wt strain (1,2) (Table 2). In total, we sequenced 14 genomes for which we obtained a mean coverage of 109x. An average of 66 ± 11 contigs per isolate were assembled.

**Table 2.** Single Nucleotide Polymorphisms (SNPs) detected in *P. aeruginosa* control and persister variants.

Contig	Position	Type	Wild type	mutant	Sample variant	Region	EFFECT	gene
contig-03	97212	complex	CGTT	AACG	6	Promotor		Promotor (Dihydrolipoyllysine-residue acetyltransferase component of pyruvate dehydrogenase complex)
contig-04	204656	snp^a^	T	G	6	CDS^d^	missense_variant c.1255A > C p.Met419Leu	Gamma-glutamylputrescine synthetase PuuA
contig-04	204666	snp	T	G	6	CDS	missense_variant c.1245A > C p.Glu415Asp	Gamma-glutamylputrescine synthetase PuuA
contig-04	204675	snp	C	T	6	CDS	synonymous_variant c.1236G > A p.Glu412Glu	Gamma-glutamylputrescine synthetase PuuA
contig-04	204719	snp	T	A	2,3,4,6,8,9,10,11,12	CDS	missense_variant c.1192A > T p.Met398Leu	Gamma-glutamylputrescine synthetase PuuA
contig-05	326236	snp	T	C	2,3,4,6,8,9,10,12,13,14	Promotor		hypothetical protein
contig-11	220551	snp	G	C	5	CDS	missense_variant c.727C > G p.Arg243Gly	hypothetical protein
contig-11	220546	del^b^	ACGCCGCCCCC	A	6	CDS	frameshift_variant c.722_731delGGGGGCGGCG p.Gly241fs	hypothetical protein
contig-13	6466	snp	G	A	11,12	CDS	synonymous_variant c.1698C > T p.Asn566Asn	hypothetical protein
contig-24	6439	complex	AGGAG	GGGAA	6	CDS	synonymous_variant c.252_256delCTCCTinsTTCCC p.87	hypothetical protein
contig-24	6457	complex	GG	AGA	6	CDS	frameshift_variant&missense_variant c.237_238delCCinsTCT p.Arg80fs	hypothetical protein
contig-24	6466	snp	G	A	6	CDS	stop_gained c.229C > T p.Arg77*	hypothetical protein
contig-24	6470	ins^c^	G	GGC	6	CDS	frameshift_variant c.224_225insGC p.Asp75fs	hypothetical protein
contig-30	53663	ins	C	CA	8	CDS	frameshift_variant c.716dupA p.Leu240fs	L-threonine 3-dehydrogenase
contig-44	1027	snp	A	G	3,9,10,11,12	CDS	missense_variant c.124A > G p.Met42Val	hypothetical protein

Notes: Data indicate the location and the contig in the wt strain and all the characteristics of the identified SNPs. The sequenced bacterial variants where the SNPs were detected are represented as numbers: 2, 3, 4, 5 and 6 correspond to wt strain grown without ciprofloxacin (CIP); variants 7, 8, 9 and 10 correspond to persister cells grown with CIP; plates 11 and 12 were grown in the presence of CIP and mannitol; and finally, 13 and 14 were grown with mannitol only.

^a^ Single nucleotide polymorphism.

^b^ Deletion.

^c^ insertion

^d^ Protein coding sequence.

**Figure 6.**
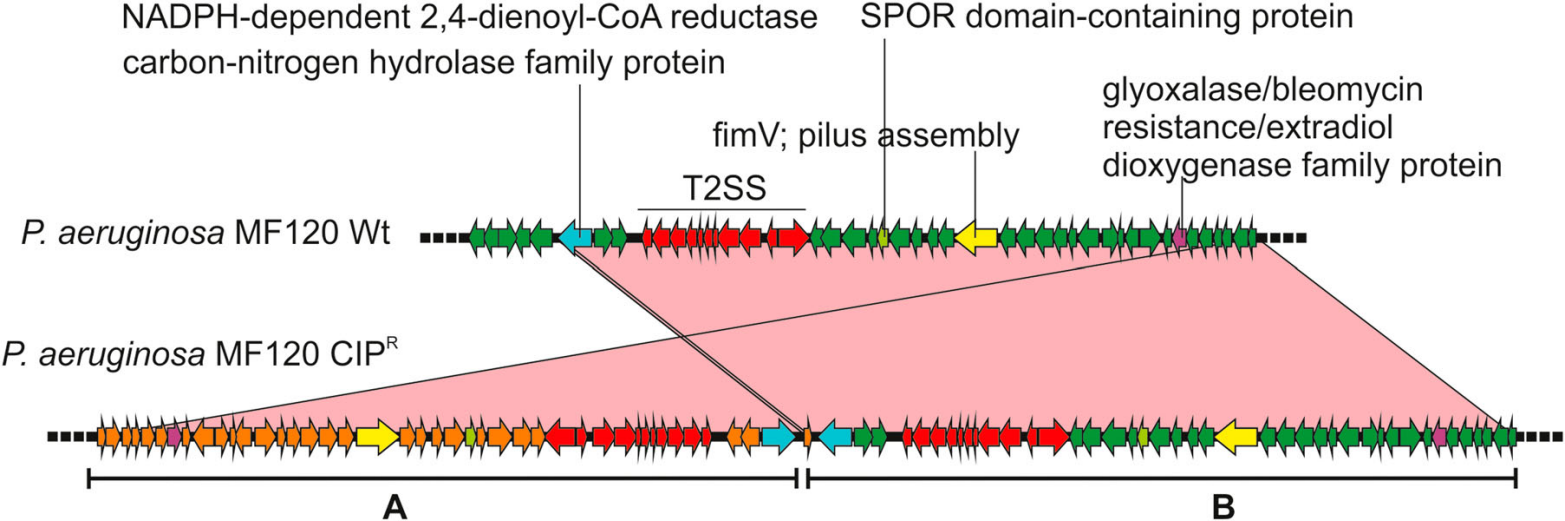
Genomic duplication in the *P. aeruginosa* CIP^R^ variant. The wild-type strain (wt) and the persister colony from a CIP-treated plate were sequenced and compared (using blastn). A region spanning 42 genes was found duplicated and inverted in the CIP^R^ variant. This is marked as A whereas the original sequence is shown as B. Relevant genes are coloured differently and their names indicated. The sequence identity of the alignment was 100% for all genes. T2SS, type 2 secretion system.

We detected a duplication in one of the colonies isolated from the plate with CIP (isolate 7). The duplicated region was 43.6 Kbp long and included 42 ORFs (Table 2 and Figure 6). Among these, the annotation revealed the presence of two genes involved in pilus assembly (*fimV* and *pilE*) and nine genes coding for general secretion pathways (type II secretion system). Interestingly, this secretion pathway is related with membrane transport during biofilm formation.

In addition, we studied single-nucleotide polymorphisms (SNPs) in the different variants, using the wt strain genome as a reference. In the colonies grown with CIP, we detected an insertion (C→CA) in the *tdh* gene (L-threonine 3-dehydrogenase) in the bigger colony and a missense substitution (A→G) in a gene coding for a hypothetical protein. Additionally, we found a mutation (T→C) in the promoter region of a hypothetical protein. The rest of detected SNPs are shown in Table 2. Thus, we did not detect any polymorphisms that could be uniquely and consistently associated with persister colonies.

## Discussion

Bacteria growing in biofilms are highly associated with increased resistance to conventional antibiotics, resulting in treatment failure [46]. Our data show the importance of evaluating biofilm formation capacity for each strain individually, as the functional characteristics of the biofilms change substantially depending on the isolate. In addition, we show that growing conditions like initial inoculum concentration and above all culture media have a dramatic effect on the resulting biofilm phenotype, with some strains even failing to form biofilms at all depending on the medium. This emphasizes the need for standardizing growing conditions and reducing the manipulation of biofilms in order to obtain consistent and reliable measures of biofilm growth and resistance [7,31]. The data presented in the current study show the large potential of impedance measures to study biofilm dynamics, for example by evaluating antibiotic resistance of individual strains and by identifying the emergence of persister cells. The results were confirmed by the counting of viable *P. aeruginosa* cells, as well as by biofilm mass quantification after crystal violet staining. Hence, Real-Time measurements of biofilm growth are reliable and could therefore be useful in providing faster assays of antibiotic susceptibility compared to more traditional end-point measures.

In the current study, the exposure of *P. aeruginosa* biofilms to conventional drugs showed that antibiotics which target bacterial cell wall synthesis such as CAZ, IPM, MEM or TZP were not able to completely prevent biofilm formation or disrupt mature pseudomonal biofilms (Figures 3 and 4). Given that none of the tested CST concentrations were able to halt the development of *P. aeruginosa* biofilms completely, we hypothesize that this antibiotic is not able to penetrate into deep biofilm layers or might alter the production of exopolysaccharide matrix compounds as observed elsewhere [21,47]. The largest anti-biofilm effect (in both prevention and eradication) was observed with CIP (an inhibitor of the DNA gyrase) and TOB (inhibitor of protein synthesis). Our data show that certain concentrations of both CIP and TOB resulted in the appearance of dormant cell populations within mature *P. aeruginosa* biofilms, in agreement with the results recently described by Soares et al. We investigated whether the use of mannitol alone or in a combination with CIP could help to eradicate mature biofilms in the clinical strain MF120, following the work of Barraud and colleagues, that detected an increase in TOB efficiency up to 3 orders of magnitude by altering bacterial metabolic activity [48]. Our results indicate that mannitol alone has neither antimicrobial nor biofilm dispersal properties, but this compound was able to enhance the efficiency of subinhibitory CIP concentrations. Specifically, mannitol at 3200 mg/L in combination with CIP (0.25 mg/L) completely killed all bacterial cells embedded in mature clinical MF120 biofilms, while 320 mg/L of mannitol combined with CIP resulted in five orders of magnitude decrease in viable cell counts compared to that of CIP alone (dose-dependent quinolone potentiation) (Figure 4(c)). We therefore suggest the possible adjuvant use of mannitol to successfully disrupt and kill antibiotic-resistant *P. aeruginosa* biofilms. Our results also show that mannitol can revert persister cells into actively growing cells faster than LB alone (Figure 5), in agreement with the proposed mechanism for this compound in bacterial metabolism [48,49].

There are several genes whose activity have been linked to the persistent phenotypes of *P. aeruginosa* [44,50]*.* However, the whole-genome sequence analysis of different phenotypical variants did not show mutations in any of these genes. Some phenotypical variants showed two genomic mutations compared to the wt strain, and future studies should demonstrate if any of these changes are responsible for any of the morphological or behavioural changes of persisters. For example, a genomic inversion has been shown in *S.aureus* to induce a phenotypic change towards small colony variants associated with persistent infections [51]. However, both the genomic inversion and the identified single nucleotide substitutions were not common to all persisters (Table 2). This suggests that, although some of these mutations could contribute to the persister phenotype, the main origin for the switch from a wt to an antibiotic tolerant, persister cell, is probably a transcriptomic change [52,53]. Thus, future work should focus on studying the transcriptional changes associated with persister populations, in order to identify potential drug targets.

We conclude that impedance-based technology could therefore help to prognose the response of a given strain to an antibiotic as well as to select the best treatment strategy for each patient individually. In the case of persister cell eradication, we show that a compound with the ability to restore bacterial metabolic activity, as it is the case with mannitol for *P. aeruginosa* biofilms, produced a dramatic improvement of the antibiotic efficacy in our system. We hope that the current study stimulates further work to test this kind of combined treatments *in vivo*.

## Supplementary Material

Editable_Supplementary_Material_Ziemyte_et_al.docx

## Data Availability

The data that support the findings of this study are available from the corresponding author upon reasonable request.
