# Supplementary material for: Real-time monitoring of Pseudomonas aeruginosa biofilm growth dynamics and persister cells’ eradication
Source: Emerg Microbes Infect. 2021 Nov 10;10(1):2062–75. doi: 10.1080/22221751.2021.1994355 (PMC8583918; doi:10.1080/22221751.2021.1994355)
Supplement: Editable_Supplementary_Material_Ziemyte_et_al.docx [file TEMI_A_1994355_SM1442.docx]

| Strain | Description/Use | Reference |
| --- | --- | --- |
| PAO1 | Laboratory strain; wt | Pseudomonas Genetic Stock Center |
| PAO1 ∆*rhl* ∆l*as* | Laboratory strain; lasI-rhlI double mutanto f PAO1 | ^1^ |
| ATCC27853 | Model strain to test antibiotic susceptibilities | ^2^ |
| MF116 | Isolate from rectal swab | This study |
| MF117 | Isolate from urinary tract infection | This study |
| MF118 | Isolate from wound exudate | This study |
| MF119 | Isolate from ulcer exudate | This study |
| MF120 | Isolate from urinary tract infection | This study |
| MF121 | Isolate from ulcer exudate | This study |
| MF122 | Isolate from wound exudate | This study |
| MF123 | Isolate from bronchial aspirate | This study |
| MF124 | Isolate from blood culture | This study |

# S1 Table. Bacterial strains used in this study.

# S2 Table. Minimum inhibitory concentration (MIC) values of ciprofloxacin (CIP), tobramycin (TOB), ceftazidime (CAZ), colistin (CST), piperacillin-tazobactam (TZP), imipenem (IPM), and meropenem (MEM) in different *P. aeruginosa* strains as measured by standard protocols and expressed as mg/L. S and R in the table indicate if the strain is considered susceptible or resistant according to EUCAST guidelines

| **Minimum Inhibitory Concentration** | | | | | | | |
| --- | --- | --- | --- | --- | --- | --- | --- |
| **Strain** | **CIP** | **TOB** | **CAZ** | **CST** | **TZP** | **IPM** | **MEM** |
| PAO1 | 0.094 **S** | 1.5 **S** | 1 **S** | 1 **S** | 3 **S** | 1.5 **S** | 0.38 **S** |
| PAO1∆*rhl* ∆*las* | 0.094 **S** | 2 **S** | 1 **S** | 0.5 **S** | 4 **S** | 2 **S** | 0.38 **S** |
| ATCC27853 | 0.125 **S** | 1 **S** | 1 **S** | 1 **S** | 4 **S** | 3 **S** | 0.25 **S** |
| MF116 | 2 **R** | **>**8 **R** | 16 **R** | 1 **S** | >64 **R** | >16 **R** | >32 **R** |
| MF117 | ≤0.5 **S** | ≤2 **S** | 8 **S** | 8 **R** | ≤8 **S** | ≤2 **S** | ≤1**S** |
| MF118 | >2 **R** | 4 **S** | 16 **R** | 1 **S** | >4 **R** | 32 **R** | 16 **R** |
| MF119 | >2 **R** | >8 **R** | **>**16 **R** | ≥2 **S** | >64 **R** | >8 **R** | ≤1 **S** |
| MF120 | 0.125 **S** | ≤2 **S** | 2 **S** | 1 **S** | ≤8 **S** | ≤ 1**S** | ≤1 **S** |
| MF121 | >2**R** | >8 **R** | 4 **S** | 1 **S** | ≤8 **S** | 4 **S** | ≤1 **S** |
| MF122 | ≤0.5**S** | ≤2 **S** | 2 **S** | ≤2 **S** | ≤8 **S** | ≤1**S** | ≤1 **S** |
| MF123 | ≤0.5**S** | ≤2**S** | ≤1 **S** | ≤2 **S** | ≤ **S** | ≤1**S** | ≤1 **S** |
| MF124 | 0.19S | 1S | 2 **S** | 1 **S** | 8 **S** | 2 **S** | 0.75 **S** |

**Table S3. Minimum inhibitory concentration (MIC)** values of ciprofloxacin (CIP), tobramycin (TOB), ceftazidime (CAZ), colistin (CST), piperacillin-tazobactam (TZP), imipenem (IPM), and meropenem (MEM) for *P. aeruginosa* MF120 persister cells grown in the presence of ciprofloxacin (final concentration 0.25 mg/L) vs MICs of persister cells re-inoculated in fresh LB medium, LB medium supplemented with mannitol (3.200 mg/L) or CIP (0.25 mg/L) for 96h. MICs were measured by standard E-test protocols. S and R in the table indicate if the strain is considered susceptible or resistant according to EUCAST guidelines. Data from two replicates for each treatment are shown.

| **Minimum Inhibitory Concentration** | | | | | | | |
| --- | --- | --- | --- | --- | --- | --- | --- |
| **Strain** | **CIP** | **TOB** | **CAZ** | **CST** | **TZP** | **IPM** | **MEM** |
| Control | 0.19 (S)  0.19 (S) | 1.5 (S)  1.5 (S) | 1 (S)  1.5 (S) | 1 (S)  2 (S) | 6 (S)\  6 (S) | 2 (S)  1.5 (S) | 0.38 (S)  0.25 (S) |
| Persisters | 2 (R)  3 (R) | 0.5 (S)  0.75 (S) | 0.75 (S)  0.75 (S) | 0.75 (S)  0.75 (S) | 3 (S)  4 (S) | > 32 (R)  > 32 (R) | 0.5 (S)  0.75 (S) |
| Persisters reinoculated in LB | 3 (R)  2 (R) | 1.5 (S)  1.5 (S) | 1 (S)  1.5 (S) | 1 (S)  1 (S) | 6 (S)  6 (S) | >32 (R)  8 (S) | 0.5 (S)  0.25 (S) |
| Persisters reinoculaed in LB + mannitol (revertant) | 0.25 (S)  0.19 (S) | 1.5 (S)  1.5 (S) | 1.5 (S)  1.5 (S) | 1 (S)  1 (S) | 8 (S)  8 (S) | 1.5 (S)  2 (S) | 0.38 (S)  0.25 (S) |
| Persisters  reinoculated to LB + CIP | 3 (R)  3 (R) | 1 (S)  0.75 (S) | 1.5 (S)  48 (R) | 1 (S)  1 (S) | 3 (S)  256 (R) | > 32 (R)  > 32 (R) | 1 (S)  4 (S) |


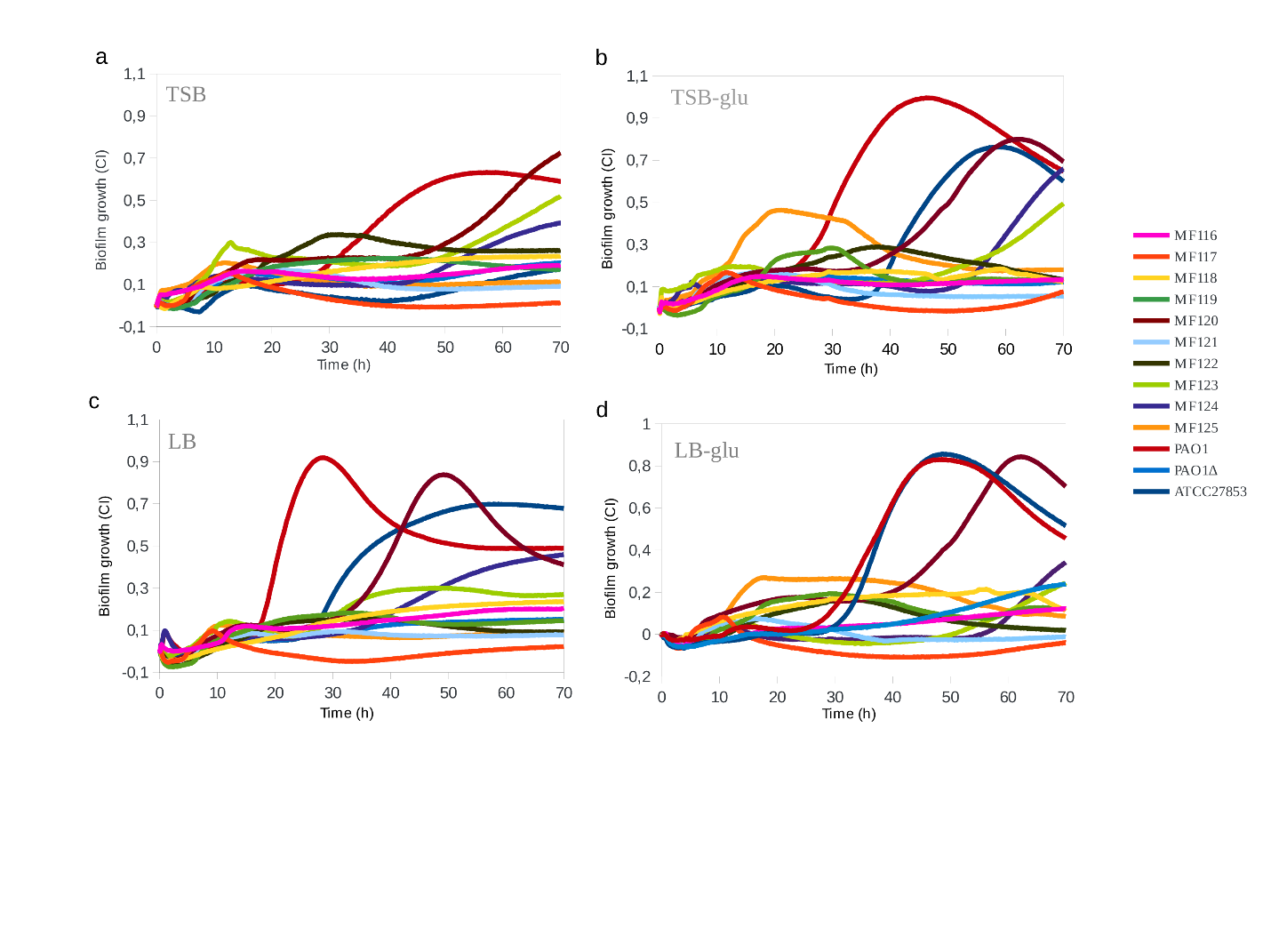


**Figure S1**. The effect of culture medium composition on *P. aeruginosa* biofilm formation: (**a)** TSB with no additional sugars; (**b)** TSB + 0.5% of glucose; (**c)** LB with no additional sugars; (**d)** LB + 0.5% of glucose. Cell Index (CI) was measured using xCELLigence equipment for 72h and correlates with total biofilm mass. Data are the means of 3 biological replicates. SDs are not shown for clarity.

#
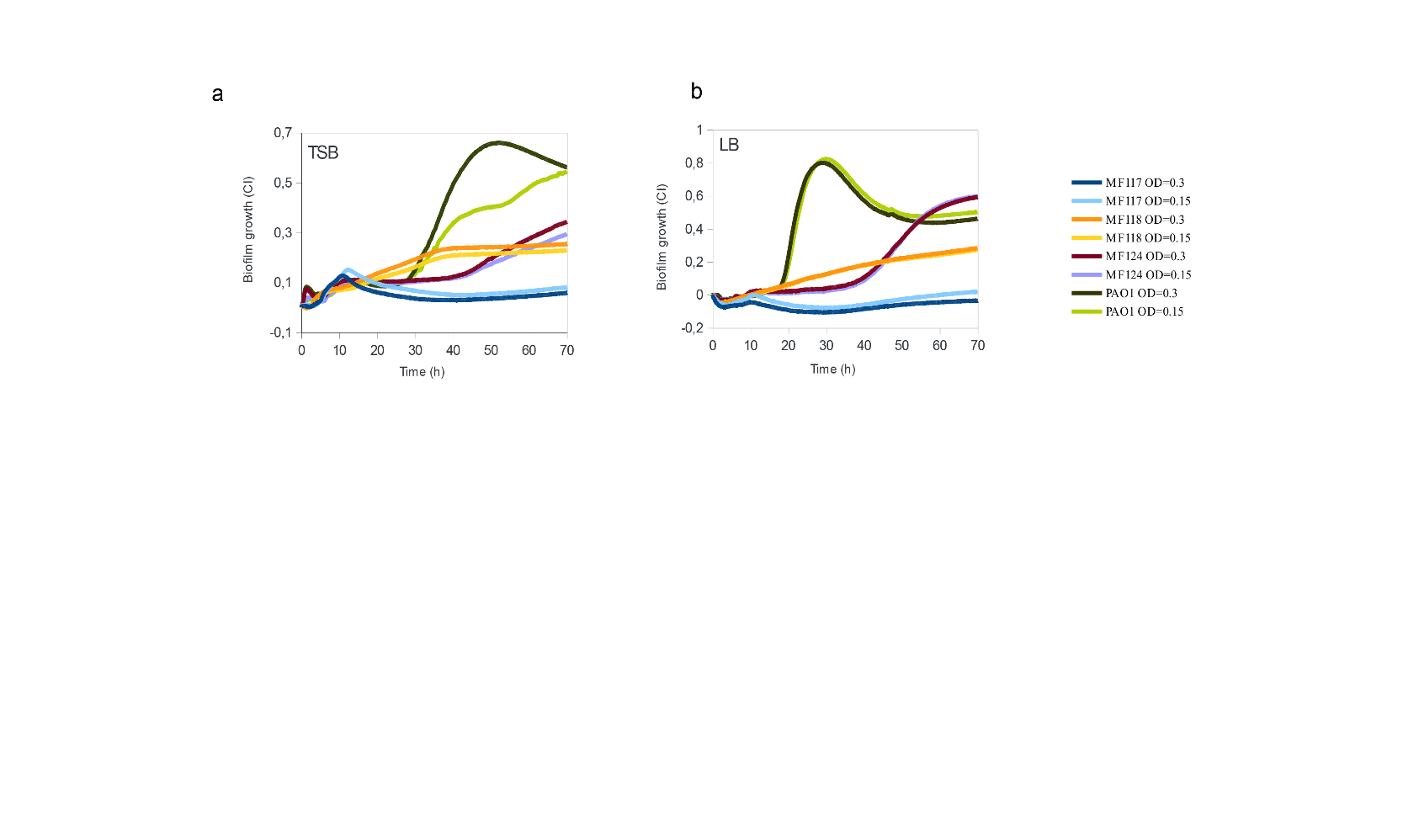


# Figure S2. The effect of initial bacterial cell optical density on *P. aeruginosa* biofilm formation in the real-time cell analysis xCELLigence system. Biofilm growth was measured using TSB and LB culture media without additional sugars every 10 minutes for 70h using initial OD of 0.15 and 0.3, respectively. Data are means of three biological replicates. SDs are not shown for clarity.

#
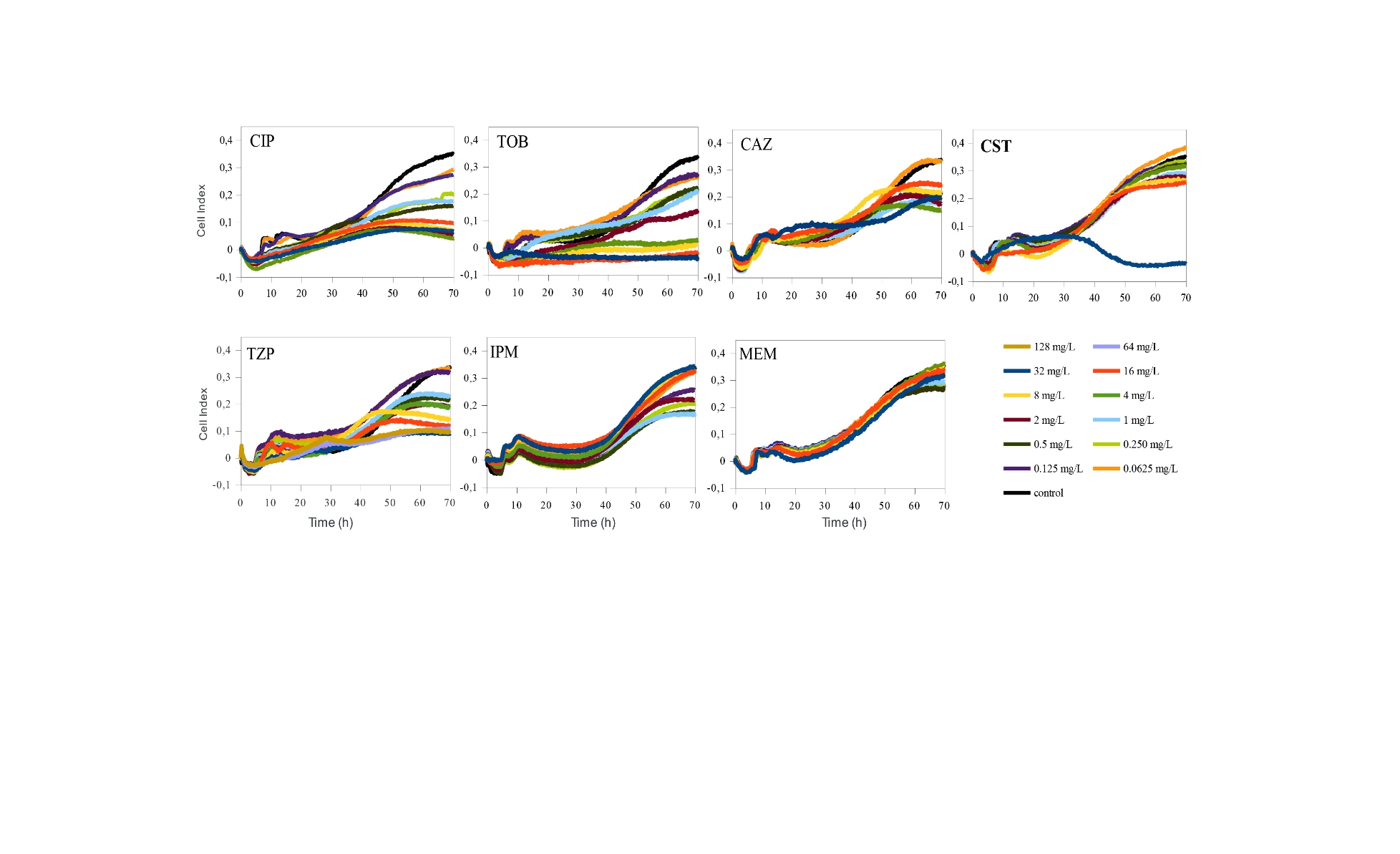


# Figure S3. Effect of ciprofloxacin (CIP), tobramycin (TOB), ceftazidime (CAZ), colistin (CST), piperacillin-tazobactam (TZP), imipenem (IPM) and meropenem (MEM) on *P. aeruginosa* MF124 biofilm formation. Graphs show estimates of total biofilm mass as quantified by impedance-based measurements. Black lines indicate untreated controls. Each line represents the mean of two replicates. All antibiotics were added at the beginning of the experiment together with bacterial inoculum, with concentrations ranging from 0.625 mg/L to 32 mg/L for all antibiotics except TPZ (0.625 mg/L to 128 mg/L). Biofilm growth was registered every 10 mins for 70h. SDs are not shown for clarity.

# References

1 Pearson JP, Pesci EC, Iglewski BH. Roles of Pseudomonas aeruginosa las and rhl quorum-sensing systems in control of elastase and rhamnolipid biosynthesis genes. *J Bacteriol* 1997; **179**. doi:10.1128/jb.179.18.5756-5767.1997.

2 Cao H, Lai Y, Bougouffa S, Xu Z, Yan A. Comparative genome and transcriptome analysis reveals distinctive surface characteristics and unique physiological potentials of Pseudomonas aeruginosa ATCC 27853. *BMC Genomics* 2017; **18**. doi:10.1186/s12864-017-3842-z.
